# Possibilities of advanced practices and Professional Master's Programs in Nursing

**DOI:** 10.1590/0104-1169.0000.2470

**Published:** 2014

**Authors:** Maria Lúcia do Carmo Cruz Robazzi

**Affiliations:** Associate Editor of the Revista Latino-Americana de Enfermagem, Coordinator of the Professional Master's Program in Nursing Technology and Innovation and Full Professor of the University of São Paulo at Ribeirão Preto College of Nursing, WHO Collaborating Centre for Nursing Research Development, SP, Brazil. E-mail: avrmlccr@eerp.usp.br

Nursing is the profession of giving care; assisting human beings is its paramount objective, with care being offered to individuals, families and community groups, aiming for health promotion, disease prevention and recovery. The care practice represents going beyond a commercial activity and can be distinguished if it produces scientific knowledge and relevant nursing care technologies^(^ 1 ^)^, thus provoking advances for the profession.

The Professional Master's Programs are a *Stricto Sensu *graduate education modality aimed at professionals who are active in the job market, granting them opportunities to propose solutions based on research, in view of the problems that exist in their daily activities, thus producing research related to the praxis, without a loss of methodological rigor. In Brazil, this modality has received approval and recognition from the Coordination for the Improvement of Higher Education Personnel (CAPES), the Brazilian agency that works towards the expansion and consolidation of *Stricto Sensu *graduate education within the national territory.

The peculiarity of this graduate education is the emphasis on studies and techniques, processes or themes that respond to the demands of the job market, focused on the performance of a high professional qualification level. Its objective is to contribute to the Brazilian productive sector in order to provide the companies and organizations with a higher level of competitiveness and productivity, whether in the public or private sector. Its graduates hold the same degree and prerogatives as holders of Academic Master's degrees; the defense of the thesis/course conclusion research should be linked to the actual problems of the professional-student's activity area^(^ 2 ^)^. 

What Nursing is concerned, when one seeks this characteristic in Master's programs is English-language countries, some similarities with the Academic Master's programs are found, adding that the Professional Master's programs emphasize teaching on themes directed at professional practices^(^ 3 ^)^. In Brazilian Nursing, the Professional Master's education is a new modality; among the 96 graduate courses recommended and recognized by the Nursing Area of CAPES, 15 are of this type, corresponding to 15.62% of all 96 courses in the Area^(^ 4 ^)^. 

In some of the productions from 2010-2012, developed in the Professional Master's programs, various protocols related to Nursing care practice were identified, focused on hemotherapy care, prevention of ventilator-associated pneumonia, technologies in intravenous therapy, efficacy of the cardiac pump, training to implement the nursing care systemization, use of papain gel in venous ulcers, care for patients submitted to puncture aspiration, welcoming for users submitted to digestive endoscopy, nursing care for children with cardiac disease, for infants based on non-pharmacological methods, among others^(^ 5 ^)^. 

Protocols are important instruments to cope with concrete practical problems, based on studies validated based on premises of scientific evidence(6). When elaborated and proposed by nurses, students in Professional Nursing Master's programs in Brazil, they can support the advancement of care practice, allowing them to obtain greater satisfaction and professional autonomy and, consequently, reverting into better health care quality for the population in the future.
